# The role of childhood adversities, *FKBP5*, *BDNF*, *NRN1*, and generalized self-efficacy in suicide attempts in alcohol-dependent patients

**DOI:** 10.1007/s43440-020-00080-8

**Published:** 2020-03-10

**Authors:** Dominika Berent, Bożena Szymańska, Dominika Kulczycka-Wojdala, Marian Macander, Zofia Pawłowska, Marcin Wojnar

**Affiliations:** 1 Masovian Regional Psychiatric Hospital Drewnica, Ząbki, Poland; 2 Central Scientific Laboratory, Medical University of Lodz, Lodz, Poland; 3 Aviation Patophysiology and Safety Flight Department, Military Institute of Aviation Medicine, Warsaw, Poland; 4 Department of Psychiatry, Medical University of Warsaw, Warsaw, Poland; 5 Department of Psychiatry, University of Michigan, Ann Arbor, MI USA

**Keywords:** Childhood adversities, Neuroplasticity, Generalized self-efficacy, Suicide attempt, Alcohol dependence

## Abstract

**Background:**

Alcohol-dependent (AD) patients report higher number of adverse childhood experiences (ACEs), develop poor social skills, and have a higher rate of suicide attempts than the general population. We hypothesize that the association between ACEs and lifetime suicide attempts in AD patients is mediated by generalized self-efficacy and selected functional single nucleotide polymorphisms (SNPs) in genes involved in the stress response and neuroplasticity, including: *FKBP5* rs1360780, *BDNF* rs6265, and *NRN1* rs1475157.

**Methods:**

176 AD patients and 127 healthy controls self-reported ACEs with the ACE Study questionnaire and three additional questions that inquired about ACE categories of acute stress; generalized self-efficacy—with the Generalized Self-Efficacy Scale. Genotyping for the three analysed SNPs was performed according to the manufacturer’s standard PCR protocol. Hypotheses were tested with bivariate analyses, multiple regression model, and mediation models.

**Results:**

Higher levels of generalized self-efficacy were associated with a blunted effect of ACEs on the risk of suicide attempts. The prevalence of the three analyzed SNPs genotypes and alleles did not differ between AD patients with a positive vs. negative lifetime history of suicide attempt and was not associated with GSES scoring.

**Conclusions:**

Generalized self-efficacy should be considered as a target for psychotherapeutic interventions aimed at reducing the risk of suicide attempts in AD patients who were exposed to childhood victimization. The negative results concerning the hypothesized role of the three analysed SNPs should be carefully interpreted due to the relatively small study sample, but represent a theoretical foundation for further research studies with larger study samples.

**Electronic supplementary material:**

The online version of this article (10.1007/s43440-020-00080-8) contains supplementary material, which is available to authorized users.

## Introduction

Alcohol-dependent (AD) patients are of concern to psychiatrists, psychotherapists, and general practitioners due to their risk of developing various health-harming behaviors. These health-harming behaviors may start as impaired diet and physical activity, leading to concomitant addictions and inefficient treatment compliance, and resulting in the development of poor social skills, poor coping with life dimensions, suicide attempts, and completed suicides [1–3]. ACEs were found to be associated with lifetime suicide attempts in a cross-sectional observation and shown to be a risk factors for lifetime suicide attempts in longitudinal ACE Study observation in the US general population [4]. Moreover, participants in the ACE study who reported at least four ACE categories were seven times more likely to suffer from alcoholism as compared to persons who reported zero ACE categories [5]. In the US and GB, national surveys in the general population showed that at least one ACE identified with the ACE Study questionnaire was reported by 64% and 46.4% of respondents, respectively [5, 6]. When a clinical sample of Polish AD patients (*n* = 196) completed the ACE Study questionnaire, over 86% confirmed exposure to at least one ACE [3]. Sexual abuse, defined as sexual penetration or other behaviors aimed at sexual satisfaction against will, was significantly associated with lifetime suicide attempts among Polish AD inpatients (*n* = 386; OR = 2.52) [7].

As reported by Nock et al. [8], lifetime prevalence of a suicide attempt was found in 2.7% of 84,850 adults from the general population across 17 countries worldwide. In same study, individuals with mental disorders showed a higher lifetime prevalence of a suicide attempt and other suicidal behaviors (e.g., ideation, plan) than the general population (mood disorders OR = 3.4–5.9; impulse-control disorders OR = 3.3–6.5; anxiety disorders OR = 2.8–4.8; substance use disorders OR = 2.8–4.6) [8]. The prevalence of suicide attempts in Poland is similar to prevalence in other European countries but higher than in the US (17.0 vs. 12.3 per 100,000) [9]. The lifetime prevalence of a suicide attempt has been shown to reach 31.9–43% in inpatient AD treatment programs in Poland [7, 10, 11].

AD patients were found to develop deficits in social cognitive abilities including emotion recognition and the ability to attribute mental states (e.g., intentions, feelings, beliefs) to others, as well as, explain and predict others’ behavior based on this information [2]. These difficulties can adversely contribute to interpersonal difficulties when someone is facing a demanding problem or confronted with an opposing individual. Together, these detailed items are assessed to evaluate generalized self-efficacy. Generalized self-efficacy is a measure of social skills that reflect one’s belief in their own resourcefulness and coping abilities to solve demanding and unforeseen situations [12]. Generalized self-efficacy is considered to be a relatively stable personality trait that is shaped in childhood and adolescence [13]. Generalized self-efficacy may be both impaired and strengthened by early stress as a part of children’s posttraumatic growth after childhood victimization, understood by developing better social skills, e.g., empathy, behavioral flexibility [14]. While comparing neuroimaging scans of children with a history of early caregiving neglect and children with no history of maltreatment, differences in the volume of hippocampi and amygdala were found. These brain structures are associated with cognition and emotion, and may partially mediate the development of future social skills [15]. The effect of ACEs on general physical and mental health in adulthood is not absolute, and siblings raised in the same household vary in coping skills, the development of alcohol and drug dependence, and risk of suicide attempts [16]. Expected environmental factors that may protect a child from a dysfunctional household include change of place of living, change in family structure, and psychological support. However, gene × environment (G × E) studies have provided data that support the hypothesis that susceptibility to the devastating health effects of ACEs throughout the lifespan may be determined by genes encoding proteins that are engaged in memory formation, neuroplasticity, and response to stress, thereby creating an endogenous source of coping skills with life dimensions [11, 17–20]. For the purpose of the present study, we chose to analyze three functional single nucleotide polymorphisms (SNPs): *FKBP5*, *BDNF*, and *NRN1.*

*FKBP5* encodes FK506-binding protein 51 (FKBP51), which is a critical controller of the stress response via hypothalamic–pituitary–adrenal (HPA) axis modulation. FKBP51 binds to and inhibits glucocorticoid receptors (GR), making FKBP51 an important moderator of GR sensitivity, and an important element of HPA axis modulation [21]. The minor homozygotes of rs1360780 are associated with a stronger induction of *FKBP5* mRNA by cortisol [22]. A meta-analysis by Wang et al. [23] showed that individuals who carry the T allele of rs1360780 and are exposed to early-life trauma had a higher risk of depression or posttraumatic stress disorder (PTSD). Among patients with recurrent depressive disorder, rs1360780 TT homozygotes reported more depressive episodes, but they also responded better to treatment with antidepressants [22]. *FKBP5* in relation to risk for suicide attempt and completed suicide was assessed in several studies with diverse results [17, 24].

*BDNF* encodes brain-derived neurotrophic factor (BDNF), which promotes the survival and differentiation of select neurons in the peripheral and central nervous systems during development, and is a major regulator of synaptic transmission and plasticity at adult synapses in many regions of the central nervous system (CNS). *BDNF* rs6265 (Val66Met, G/A) is a missense functional polymorphism. Met allele carriers who have been exposed to childhood abuse have been shown to have the lowest levels of serum BDNF and smaller hippocampal volumes [25, 26]. Plasma levels of BDNF were also lower in suicidal depressed patients when compared to non-suicidal depressed subjects [27, 28]. In contrast, a post-mortem study found no association between *BDNF* rs6265 and suicide among a sample of 181 autopsied suicide victims with a history of alcohol and/or drug addiction [29].

*NRN1* encodes neuritin-1, which functions extracellularly to modulate neurite outgrowth and synapse maturation. Few studies have examined the *NRN1* rs1475157 functional polymorphism, but it was assessed in a study using a non-clinical sample to evaluate its role in developing sub-depressive symptoms [30].

To our knowledge, this is the first study to test the following three hypothetical mediational models in AD patients: (1) a model wherein generalized self-efficacy mediates the relationship between childhood victimization (measured as a sum of 13 possible ACEs categories) and positive lifetime history of a suicide attempt; (2) a model wherein *FKBP5* rs1360780, *BDNF* rs6265, and *NRN1* rs1475157 genes and alleles mediate the relationship between childhood victimization (measured as a sum of 13 possible ACEs categories) and a positive lifetime history of suicide attempt in AD patients; (3) a model wherein *FKBP5* rs1360780, *BDNF* rs6265, and *NRN1* rs1475157 genes and alleles mediate the relationship between childhood victimization (measured as a sum of 13 possible ACEs categories) and generalized self-efficacy.

## Materials and methods

### Sample and ethical statement

AD patients and controls gave written informed consent to participation in the study. Participants were informed in the study consent form that they have the right to withdraw consent at any step of the study without giving any reason, and participants were ensured confidentiality of the provided information. The study was approved by the Local Bioethics Committee: nos. RNN/467/13/KB and KB/843/13/P. The study was carried out in accordance with the ethical standards laid down in the 1964 Declaration of Helsinki and its later amendments. In the current study, controls were introduced to compare the distribution of alleles in *FKBP5* rs1360780, *BDNF* rs6265, and *NRN1* rs1475157 genes between control subjects and AD patients.

AD patients aged ≥ 18 were recruited from inpatient AD treatment programs located in Central Poland. Potentially eligible patients had a clinically established diagnosis of AD by at least two psychiatrists according to the ICD-10 (F10.2) diagnostic criteria [31] and were admitted for addiction psychotherapy or for treatment of alcohol withdrawal syndrome. AD patients were invited to participate in the study at least 1 week from the beginning of hospitalization to exclude patients with acute withdrawal syndrome at the time of the study. Of the 209 patients who gave informed consent to participate in the study, 33 did not undergo further analysis because of the following exclusion criteria (of note, some individuals fit multiple exclusion criteria): (1) co-occurring psychiatric disorder requiring current medication (*n* = 6); (2) returning an uncompleted study questionnaire (*n* = 30); (3) consent withdrawal, primarily due to finding the questions too intimate or time-consuming (*n* = 3); (5) prior receipt of chemotherapy consisting of drugs that influence DNA methylation, i.e., 5-azacytidin and decitabine (criterion implemented for the purpose of the previous study on genome methylation (*n* = 1) [20]; and (6) insufficient quality of DNA available (*n* = 18).

The controls were healthy volunteers aged ≥ 18 from the local community. Of the 140 individuals who gave informed consent to participate in the study, 13 were not included in further analysis due to the following exclusion criteria. Exclusion criteria for controls were: (1) prior lifetime diagnosis of a mental disorder according to the ICD-10 [31] (*n* = 6); (2) prior lifetime suicide attempt or self-mutilation (*n* = 3); (3) reaching an AUDIT score [32] indicating alcohol abuse (F10.1 according to the ICD-10) [31] or possible AD (F10.2 according to the ICD-10) [31] (*n* = 6); (4) returning the study questionnaires with incomplete data (*n* = 2); (5) prior receipt of chemotherapy consisting of drugs that influence DNA methylation, i.e., 5-azacytidin and decitabine (*n* = 0); or (6) insufficient quality of DNA available (*n* = 3).

AD patients and the controls were native, unrelated inhabitants of Central Poland, recruited into the study between 2013 and 2015 and included in previous studies [11, 20].

### Measures

Sociodemographic and clinical characteristics of the study participants were identified with a structured self-report questionnaire designed for the study. The researcher remained present during the completion of the questionnaires to address the participants’ questions and to make sure the respondents understood all of the items. Lifetime history of suicide attempts was measured using the following single-item question (Yes/No): “Have you ever tried to commit suicide during your lifetime?”.

The AUDIT [32], with a Cronbach’s alpha of 0.85, was applied to characterize current alcohol intake severity in patients with AD and to exclude healthy volunteers with alcohol abuse (F10.1 according to the ICD-10) or suspected AD (F10.2) from the study [31].

Childhood and adolescent victimizations were identified with Yes/No questions about 13 categories of ACEs that occurred during the first 18 years of life. The first ten questions, developed by Kaiser Permanente and the US Centers for Disease Control and Prevention (ACE Study questionnaire) [5], consider chronic exposure to abuse (physical, verbal, and sexual abuse, or neglect) and family dysfunction (the loss of one or both parents for any reason; exposure to domestic violence between family members; and growing up in a household with mental illness, alcohol abuse, drug abuse, or incarceration). We added three more questions to the structured self-report questionnaire designed for this study to evaluate events that also took place under the age of 18 and are considered to be acute stressors, including witnessing a family member’s suicide attempt; witnessing a family member’s death due to any cause (except for completed suicide); and witnessing a stranger’s death due to any cause (e.g., traffic accident). For the purpose of our study, the list of the standard ten ACE study questions together with our three additional ACE acute stressors questions is referred to as the ‘ACE (13)’ questionnaire.

Generalized self-efficacy was measured with the Polish version of the Generalized Self-Efficacy Scale (GSES). The GSES was developed by Schwarzer, Jerusalem and Juczyński, and is a 10-item psychometric scale of internal reliability measured by the Cronbach’s alpha equal to 0.85 [33]. The scale is devoted to assessing optimistic self-beliefs into coping with a variety of difficult demands in life [33]. Responses are made on a 4-point scale for each item with total scores ranging from 10 to 40. The higher the score, the higher the individual’s generalized sense of self-efficacy. A total of ≤ 24 points is interpreted as ‘low’ generalized self-efficacy; between 25 and 29 points is considered to be ‘medium’; and ≥ 30 points is considered to be a ‘high’ score [33].

To address the possible bias associated with the participant’s intentional attempt to present him/herself in either a better or worse mental and general condition, the researcher who remained present during the completion of the questionnaire was not involved in the patients’ therapy.

### Buccal smear obtaining and laboratory testing

Laboratory testing was performed at the Central Scientific Laboratory of the Medical University in Lodz. Buccal smears were obtained by rubbing the buccal mucosa with a sterile, DNA-free set of forensic swabs (Sarstedt). The smears were obtained by trained personnel and then stored in accordance with the manufacturer’s instructions until laboratory analysis. The buccal smears were obtained at least 2 h after eating, tooth brushing, cigarette smoking, or gum chewing. Genomic DNA was isolated from the buccal swabs using a High Pure PCR Template Preparation Kit (Roche), according to the manufacturer’s protocol. DNA was eluted in 100 μl Elution Buffer and quantified using a Picodrop spectrophotometer (Picodrop Limited). The quality of the DNA samples was analyzed by measuring the ratio of absorption at 260/280 nm. Purified total DNA was immediately used for PCR reactions or stored at − 20 °C. *BDNF* rs6265, *FKBP5* rs1360780, and *NRN1* rs1475157 were analyzed using a commercially available Pre-made TaqMan SNP Genotyping Assay (Applied Biosystems, Assay ID: *BDNF* C_11592758_10, *FKBP5* C_8852038_10, *NRN1* C_8912064_20). The assay consisted of PCR primers and reporter probes that were labeled with a quencher (MGB) and either 6-carboxyfluorescein (FAM) or VIC (Applied Biosystems proprietary dye with *λ*_ex_ = 488 nm and *λ*_em_ = 552 nm). Amplification of the probe-specific product causes cleavage of the probe, thus generating an increase in reporter fluorescence. Amplification was performed according to the manufacturer’s standard PCR protocol. Briefly, 10 ng total DNA was mixed with 10 μl TaqMan Genotyping PCR Master Mix and 0.5 μl TaqMan Assay, to a final volume of 20 μl. PCR thermal cycling was performed as follows: (1) initial denaturing at 95 °C for 10 min; (2) 40 cycles of 92 °C for 15 s; and (3) 60 °C for 1 min. Thermal cycling was performed using a GeneAmp PCR System 9700 (Applied Biosystems). Each 96-well plate contained 92 test samples and four reaction mixtures without the DNA template (i.e., no-template control).

The end-point fluorescence intensities of each probe were monitored using the ABI7900HT Real-Time PCR System (Applied Biosystems). The genotypes were determined automatically and then visually verified based on the dye component’s fluorescence emission data depicted in the *X*–*Y* scatter-plot of sequence detection system 2.3 Software.

### Statistical analysis

Calculations were performed using SPSS Statistics version 25. Normality of data distribution was evaluated with the Shapiro–Wilk test.

Between-group comparisons were made by the Mann–Whitney *U* test for independent samples (i.e., differences between means) and contingency tables was compared by the Chi-square test (when the expected numbers were above 5) or the Fisher exact test (when the expected numbers were below 5). Bonferroni correction for multiple testing was applied.

Characteristics that differed significantly for AD patients with a positive and negative lifetime history of suicide attempts were further analyzed in a logistic regression model, to assess the odds ratio of a suicide attempt among AD patients.

Using the results of logistic regression, a mediation model was built, wherein the mediator between ACEs and suicide attempts was general self-efficacy, as measured with the GSES.

The level of statistical significance was set at *p* < 0.05.

Propensity score matching (PSM) with 1:1 matching of controls and AD patients based on their age range, sex, and education was set to reduce possible differences associated with these sociodemographic variables. PSM was used when we examined the distribution of *FKBP5* rs1360780, *BDNF* rs6265, and *NRN1* rs1475157 alleles and genotypes among AD patients and controls divided by low, medium, and high GSES levels. The PSM was also implemented to match AD patient subgroups with positive vs. negative lifetime history of a suicide attempt based on age range, sex, and education, and also to reduce recall bias in self-reports of ACEs categories (see Supplementary Material for PSM analysis).

## Results

### Study sample

The study analyzed 176 AD patients (134 males and 42 females) aged 43.4 ± 10.5 (mean ± SD years) and 127 healthy volunteers (96 males and 31 females) aged 39.4 ± 12.0 (mean ± SD years. At the time of the study, AD patients scored 27.2 ± 7.5 points (mean ± SD) on the Alcohol Use Disorders Identification Test (AUDIT) [32].

### *FKBP5* rs1360780, *BDNF* rs6265, and *NRN1* rs1475157 genotype allelic distribution

There were no significant differences in the distribution of *FKBP5* rs1360780, *BDNF* rs6265, and *NRN1* rs1475157 alleles and genotypes between AD patients and controls (Table 1).

**Table 1:** The distribution of *FKBP5* rs1360780, *BDNF* rs6265, and *NRN1* rs1475157 alleles and genotypes in AD patients (*n* = 176) and control subjects (*n* = 127)

	Controls (*n* = 127)		AD Patients (*n* = 176)		Chi^2^	*p*
	*n*	%	*n*	%		
FKBP5 rs1360780						
T/T	10	7.9	10	5.7		
C/T	55	43.3	63	35.8	2.882	0.237^2^
C/C	62	48.8	103	58.5		
C	117	92.1	166	94.3	0.575	0.448^2^
T	65	51.2	73	41.5	2.801	0.094^2^
BDNF rs6265						
A/A	3	2.4	1	0.6		
G/A	30	23.6	58	33.0	–	0.100^1^
G/G	94	74.0	117	66.5		
G	124	97.6	175	99.4	–	0.177^1^
A	33	26.0	59	33.5	1.983	0.159^2^
NRN1 rs1475157						
G/G	3	2.4	5	2.8		
A/G	33	26.0	43	24.4	–	0.942^1^
A/A	91	71.7	128	72.7		
A	124	97.6	172	97.7	–	0.959^1^
G	36	28.3	47	26.7	0.100	0.752^2^

*p*—level of statistical significance with Bonferroni correction

AD-alcohol-dependent

^1^Fisher’s exact test

^2^Chi square test

To search for a possible association between *FKBP5* rs1360780, *BDNF* rs6265, and *NRN1* rs1475157 alleles and generalized self-efficacy, AD patients and controls were divided by low, medium, and high GSES outcomes. There were no significant differences in the distribution of *FKBP5* rs1360780, *BDNF* rs6265, and *NRN1* rs1475157 alleles between AD patients and controls for each GSES outcome. Also, no significant differences in the distribution of *FKBP5* rs1360780, *BDNF* rs6265, and *NRN1* rs1475157 alleles were found when AD patients of low, medium and high GSES outcomes were compared, and similar results were found in controls (Table 2). In addition, no significant difference was found when the distribution of *FKBP5* rs1360780, *BDNF* rs6265, and *NRN1* rs1475157 alleles and genotypes were compared between AD patients with a positive or negative lifetime history of suicide attempts (Table 3). Thus, we did not enter *FKBP5* rs1360780, *BDNF* rs6265, and *NRN1* rs1475157 alleles and genotypes into the multiple logistic regression model for suicide attempts in AD patients (Table 4). For the expected mediation model, we did not include *FKBP5* rs1360780, *BDNF* rs6265, and *NRN1* rs1475157 alleles and genotypes as possible mediators of the relationship between ACEs and suicide attempts, as well as, between ACEs and generalized self-efficacy.

**Table 2:** The distribution of *FKBP5* rs1360780, *BDNF* rs6265, and *NRN1* rs1475157 alleles in AD patients (*n* = 176) and the controls (*n* = 127), divided by low, medium, and high GSES outcomes

	GSES	Low		Medium		High		*p*
		*n*	%	*n*	%	*n*	%	
*BDNF* rs6265								
AD patients	C	39	22.3	47	26.9	89	50.9	0.227^1^
	T	17	28.8	14	23.7	28	47.5	0.373^1^
Controls	C			10	8.1	114	91.9	> 0.999^1^
	T			2	6.1	31	93.9	> 0.999^1^
AD patients vs	C					0.260^1^		
Controls	T			0.708^1^		0.435^2^		
*FKBP5* rs1360780								
AD patients	C	39	23.5	43	25.9	84	50.6	0.527^1^
	T	16	21.9	21	28.8	36	49.3	0.872^2^
Controls	C			10	8.5	107	91.5	> 0.999^1^
	T			3	4.6	62	95.4	0.199^1^
AD patients vs	C			> 0.999^1^		0.423^2^		
Controls	T			0.494^1^		0.074		
*NRN1* rs1475157								
AD patients	A	38	22.1	46	26.7	88	51.2	0.345^1^
	G	10	21.3	14	29.8	23	48.9	0.852^2^
Controls	A			10	7.9	117	92.1	> 0.999^1^
	G			2	5.6	34	94.4	0.724^1^
AD patients vs	A			> 0.999^1^		0.635^1^		
Controls	G			0.708		0.609^2^		

*p* level of statistical significance with Bonferroni correction

*GSES* generalized self-efficacy scale, *AD* alcohol-dependent

^1^Fisher’s exact test

^2^Chi square test

**Table 3:** Comparison of AD patients (*n* = 176) with a negative vs. positive lifetime history of at least one suicide attempt

	Lifetime history of at least one suicide attempt in AD patients		*p*
	Negative	Positive	
	*n* = 108	*n* = 68	
Age	45.16 (10.14)	40.79 (10.49)	**0.006**^1^
Mean ± SD			
Gender *n* (%)			
Female	20 (18.5)	22 (32.4)	0.036^2^
Male	88 (81.5)	46 (67.6)	
Place of living *n* (%)			
Village	5 (4.6)	6 (8.8)	
Urban area	103 (9.4)	62 (91.2)	0.340^3^
Education *n* (%)			
Elementary	29 (26.9)	18 (26.5)	0.979^2^
Vocational	26 (24.1)	18 (26.5)	
High school	41 (38.0)	24 (35.3)	
University degree	12 (11.1)	8 (11.8)	
Employment status *n* (%)			
Employed	21 (19.6)	20 (29.4)	0.412^3^
Unemployed	68 (63.6)	41 (60.3)	
Retired	8 (7.5)	3 (4.4)	
On pension	10 (9.3)	4 (5.9)	
Marital status *n* (%)			
Single	42 (38.9)	27 (39.7)	0.590^2^
Married	26 (24.1)	12 (17.6)	
Divorced	28 (26.0)	23 (33.8)	
Widowed	12 (11.1)	6 (8.8)	
Living status *n* (%)			
Alone	39 (36.1)	26 (38.2)	0.195^2^
With family	55 (50.9)	27 (39.7)	
With partner	14 (13.0)	15 (22.1)	
ACEs categories number (1–10)*			
Mean ± SD	2.13 (2.11)	3.78 (2.64)	**< 0.001**^1^
ACEs categories number (1–13)**			
Mean ± SD	2.47 (2.36)	4.57 (2.89)	**< 0.001**^1^
GSES			
Mean ± SD	29.40 (5.97)	26.38 (6.64)	**0.004**^1^
*FKBP5* rs1360780 *n* (%)			
T/T	7 (6.5)	3 (4.4)	
C/T	36 (33.3)	27 (39.7)	0.642^3^
C/C	65 (60.2)	38 (55.9)	
C	101 (93.5)	65 (95.6)	0.743^3^
T	43 (39.8)	30 (44.1)	0.573^2^
*NRN1* rs1475157 *n* (%)			
G/G	4 (3.7)	1 (1.5)	
A/G	24 (22.2)	19 (27.9)	0.541^3^
A/A	80 (74.1)	48 (70.6)	
A	105 (97.2)	67 (98.5)	> 0.999^3^
G	28 (25.9)	19 (27.9)	0.861^2^
*BDNF* rs6265 *n* (%)			
A/A	0 (0)	1 (1.5)	
G/A	36 (33.3)	22 (32.4)	0.585^3^
G/G	72 (66.7)	45 (66.2)	
G	108 (100)	68 (98.5)	0.386^3^
A	36 (33.3)	23 (33.8)	> 0.999^2^

*p* level of statistical significance with Bonferroni correction. Bold values indicate statistical significance

*ACEs* adverse childhood experiences; *assessed with ACE Study questionnaire; **assessed with ACE (13) questionnaire; *AD* alcohol-dependent, *GSES* generalized self-efficacy scale, *SD* standard deviation

^1^Mann–Whitney *U* test

^2^Chi square test

^3^Fisher’s exact test

**Table 4:** Multiple logistic regression model for suicide attempts in AD patients, with age and sex as control variables

	*B*	SE	*Z*	*p*	OR	95% CI for EXP(B)		*Χ*^2^	*p*
						LL	UL		
Model 1									
ACE Study questionnaire scoring	0.36	0.08	19.88	**0.000**	1.44	1.23	1.69	36.94	**< 0.001**
GSES	− 0.07	0.03	5.30	**0.021**	0.94	0.88	0.99		
Model 2									
ACE(13) questionnaire scoring	0.42	0.08	27.14	**0.000**	1.52	1.30	1.78	49.57	**< 0.001**
GSES	− 0.08	0.03	6.78	**0.009**	0.92	0.87	0.98		

Bold values indicate statistical significance

*B* unstandardized coefficient, *SE* standard error, *Z* Wald test value, *p* level of statistical significance, *OR* odds ratio, *LL and UL* lower limit and upper limit of the 95% confidence interval for Exp(B), ^1^Chi square test, *ACE* adverse childhood experience, *GSES* generalized self-efficacy scale

For the calculations presented in Tables 2 and 3, we additionally performed a 1:1 PSM of AD patients and controls (controlling for effects of sex, age and education) and found that all differences remained the same, i.e., significant (*p *≤ 0.05) or not significant (*p *> 0.05) (see the Supplementary Material, Tables 2A and 3A, respectively).

However, these negative findings have to be carefully interpreted and require further studies with larger study samples. The proportion of samples should have the following number to be able to estimate differences at a 0.05 level of significance and at a power of 0.8: for the *FKBP5* C allele, 346 individuals are needed; *FKBP5* T allele, 272 individuals; *BDNF* G allele, 1569 individuals; *BDNF* A allele, 1085 individuals; *NRN1* A allele, 1569 individuals; *NRN1* G allele, 625 individuals.

### Bivariate comparisons between AD suicide-attempters and non-attempters, and a multiple logistic regression model predicting suicide attempts in AD patients

AD patients with a lifetime history of suicide attempts were younger, reported a higher number of ACEs [both within the ACE Study questionnaire and ACE(13) questionnaire], and scored significantly lower scores on the GSES than AD patients without a history of lifetime suicide attempts (Table 3). These associations remained significant when 1:1 PSM was implemented, wherein AD patients with positive and with a negative history of lifetime suicide attempts were matched on sex, age, and education (see the Supplementary Material, Table 3A). AD patients with a positive and negative lifetime history of suicide attempts were initially compared according to selected demographic and social variables, which are factors that may be associated with lifetime suicide attempts and assessed in studies of suicide attempts and other suicide behaviors and observed by Central Statistical Office of Poland [8, 34, 35].

At the first step, the main analyzed independent variables (i.e., ACE Study Questionnaire scoring and GSES scoring) were introduced into the logistic regression model (Model 1, Table 4). This model was checked for outliers using Cook’s distance, wherein values over three standard deviations from the mean were considered to be outliers. Four outliers were identified in this model. The model without control variables was found to be well calibrated [Hosmer–Lemeshow test (*χ*^2^ (7) = 8.44; *p* = 0.392)] and explained 17.9% of the variability in the dependent variable (Cox-Snell *R*^2^ = 0.179; Nagelkerke’s *R*^2^= 0.243). In this model, ACE Study Scoring and GSES scoring were significant independent variables (*p* ≤ 0.013). After controlling for age and sex, the model remained well calibrated [Hosmer–Lemeshow test (*χ*^2^(8) = 12.74; *p* = 0.121)] and both ACE Study Scoring and GSES scoring remained significantly associated with lifetime suicide attempts. After controlling for age and sex, the model explained 19.3% of the variability in the dependent variable (Cox-Snell *R*^2^ = 0.193; Nagelkerke’s *R*^2^ = 0.262). With each additional ACE category reported with the ACE study questionnaire, the risk of lifetime suicide attempt increased by 44%, and with each point scored on the GSES, the risk for lifetime suicide attempt was reduced by 6.4%. Control variables added to model were not significantly associated with lifetime suicide attempts (age *p* = 0.227; sex *p* = 0.335).

The same analyses were performed using a logistic regression, Model 2 (Table 4). Here, six outliers were excluded using Cook’s distance. The model that did not control for age and sex was found to be well calibrated [Hosmer–Lemeshow test (*χ*^2^(8) = 3.07; *p* = 0.930)] and explained 23.9% of the variability in the dependent variable (Cox-Snell *R*^2^ = 0.239; Nagelkerke’s *R*^2^ = 0.325). Scores on both the ACE (13) questionnaire and the GSES were significantly associated with the dependent variable (*p* ≤ 0.06). After controlling for age and sex, scores on both the ACE (13) questionnaire and the GSES remained significantly associated with lifetime suicide attempts. The model explained 25.3% of the variability in the dependent variable (Cox-Snell *R*^2^ = 0.253; Nagelkerke’s *R*^2^ = 0.344). With each ACE category reported on the ACE (13) questionnaire, the risk of lifetime suicide attempts increased by 51.9%, and with each point scored with the GSES, the risk for lifetime suicide attempts decreased by 7.6%.

### Mediation models for ACEs and suicide attempts

Due to the cross-sectional design of our study, the direction of causality cannot be undoubtedly determined. Mediation model is a statistical method that allows one to explain values of a dependent variable (here, self-reported lifetime suicide attempt) as indirectly caused by values of an independent variable (here, self-reported sum of ACEs categories), without favoring any specific statistical model or set of identifying assumptions [36].

**Fig. 1:**
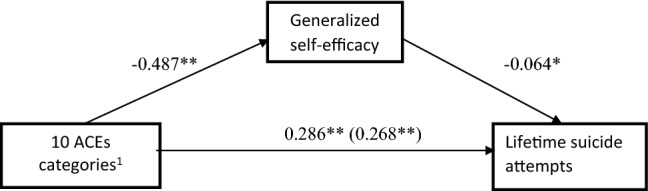
Generalized self-efficacy as a mediator of the relationship between ten categories of childhood adversities and suicide attempts in AD patients. The values in the figure represent unstandardized path coefficients. *p < 0.05; **p < 0.001. ^1^10 ACEs categories assessed with ACE Study questionnaire: ACE1 psychological abuse, ACE2 Physical abuse, ACE3 sexual abuse, ACE4 emotional neglect, ACE5 physical neglect, ACE6 contact loss with one or both parents due to separation, divorce, or other reason, ACE7 witnessing physical abuse towards one’s mother or stepmother, ACE8 problem drinking/alcoholic/street drug use of a household member, ACE9 mental illness or suicide attempt of a household member, ACE10 incarceration of a household member

**Fig. 2:**
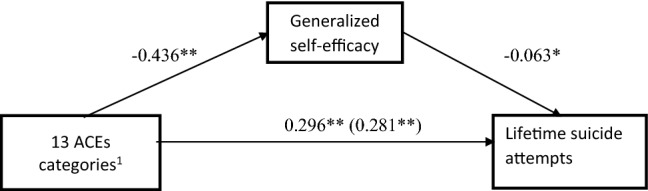
Generalized self-efficacy as a mediator of the relationship between 13 categories of childhood adversities and suicide attempts in AD patients. The values in the figure represent unstandardized path coefficients. *p < 0.05; **p < 0.001. ^1^13 ACEs categories assessed with ACE(13) questionnaire: ACE1 psychological abuse, ACE2 physical abuse, ACE3 sexual abuse, ACE4 emotional neglect, ACE5 physical neglect, ACE6 Contact loss with one or both parents due to separation, divorce, or other reason, ACE7 witnessing physical abuse towards one’s mother or stepmother, ACE8 problem drinking/alcoholic/street drug use of a household member, ACE9 mental illness or suicide attempt of a household member, ACE10 incarceration of a household member, ACE11 witnessed a family member’s suicide attempt, ACE12 witnessed a family member’s death of any cause (except for completed suicide), ACE13 witnessed a stranger’s death of any cause (i.e., a traffic accident)

We built two mediation models that included ACEs as an independent variable, measured either with the ACE Study questionnaire (Fig. 1) or with the ACE(13) questionnaire (Fig. 2). In both models, a lifetime history of suicide attempt was entered as the dependent variable and generalized self-efficacy, as measured with the GSES, was entered as a mediator. Mediation was partial in both models. That is, although generalized self-efficacy was added as a mediator, the effect of ACEs on lifetime suicide attempts remained significant in both models (Figs. 1 and 2).

Based on Fig. 1, a higher number self-reported ACEs categories (out of 10 possible categories) was associated with a significantly higher risk of suicide attempts (*p *< 0.001) and significantly lower generalized self-efficacy, as assessed with the GSES (*p * < 0.001). Lower GSES scores were associated with a significantly higher risk of suicide attempts (*p* < 0.005). Including generalized self-efficacy as a possible mediator of the relation between ACEs and suicide attempts revealed that a significant indirect effect of ACEs on suicide attempts through GSES (Sobel test, *Z* = 1.73; *p* = 0.042). In this model, mediation is partial while the direct effect remained significant (0.268; SE = 0.071; *p* < 0.001).

Figure 2 shows that a higher number self-reported ACE(13) categories (out of 13 possible categories) was associated with a significantly higher risk of suicide attempts (*p* < 0.001) and significantly lower generalized self-efficacy (*p* < 0.001). Lower scores on the GSES were associated with a significantly higher risk of suicide attempts (*p* < 0.005). The second analysis yielded a significant indirect effect of ACEs on suicide attempts through GSES (Sobel test, *Z* = 1.72; *p* = 0.043), whereas the direct effect remained significant (0.281; SE = 0.066; *p *< 0.001). Thus, GSES partially mediated the relationship between ACEs and suicide attempts.

## Discussion

Our study confirmed an association between the examined ACEs categories (physical, verbal, and sexual abuse; emotional and physical neglect; several household dysfunctions; witnessing a family member’s suicide attempt or death and witnessing stranger’s death) and lifetime suicide attempts in AD patients. The odds ratio of a suicide attempt increased by 1.44 and 1.52 with each additional self-reported ACE category, as measured by the ACE Study Questionnaire (Model 1 of Table 4) and the ACE (13) questionnaire (Model 2 of Table 4), respectively. The odds ratio of a suicide attempt decreased by 6% in Model 1 and by 8% in Model 2 (see Table 4) with each point on the GSES. When we tested the hypothetical model wherein generalized self-efficacy served as a mediator of the relationship between the sum of self-reported ACEs categories and lifetime suicide attempts in AD patients, generalized self-efficacy partially mediated the effect of ACEs on lifetime suicide attempts in the study group. We failed to find a significant association between *FKBP5* rs1360780, *BDNF* rs6265, and *NRN1* rs1475157 alleles/genotypes with generalized self-efficacy and lifetime suicide attempts in AD patients. However, due to the relatively small study sample, we suggest careful interpretation of these null results, and this topic requires further studies with larger study samples.

According to the report on suicide attempts and completed suicide in the general Polish population (accounting for 38,433,000 inhabitants in 2016) by the Central Statistical Office of Poland, 5405 of the 9861 registered suicide attempts were lethal [35, 37]. Completed suicides were more frequently seen in individuals of male sex, urban area citizens, married, currently employed, and with vocational education (10–11 years of education in Poland) (of note, data were presented as descriptive statistics only, and the population included individuals under the age of 18) [35]. The majority of suicide attempts in the general Polish population were observed in individuals between the ages of 30 and 49 years [35]. In our clinical sample of AD patients, no significant differences were observed when AD patients with positive and negative history of lifetime suicide attempt were compared according to sex, place of living (i.e., village vs. urban area), education, marital and employment status, or living alone vs. with a family member or partner. However, AD patients who confirmed a history of lifetime suicide attempt were significantly younger than AD patients with a negative lifetime history. Gerhant et al. also found no significant differences when Polish AD patients with and without a lifetime history of suicide attempts were compared according to sex, education, living area, employment, or presence of a partner [38]. That study also found that AD patients with a lifetime history of suicide attempt were significantly younger than AD patients without a lifetime history [38]. In the current study, we analyzed ten categories of ACEs based on the ACE Study questionnaire used in other studies and three more ACEs categories that included self-reported witnessing of another individual’s death or suicide attempt. It is important to underline that, although witnessing the death of a stranger for any reason (e.g., traffic accident) (ACE13) may be considered to be an acute stressor, witnessing a family member’s suicide attempt (ACE11) or death to any cause (except for completed suicide) (ACE12) is not only an acute stressor but can be associated with a loss of an attachment figure and safety. Sudden death of a family member, e.g., due to stroke, murder or suicide, is a risk factor for an impaired course of bereavement, with its lifetime devastating effects [39]. As reviewed and studied by Radziwiłłowicz, a critical number of ACEs (at least 3 according to the Radziwiłłowicz study) leads to crossing a level of risk for developing future mental disorders (e.g., depression), and this risk increases when ACEs are chronic [39, 40]. Witnessing a stranger’s death due to any cause (ACE13) is an acute and accidental, and potentially traumatic experience, which was shown to be of lower significance in risk for PTSD than personal victimizations, according to the US National Survey of Adolescents [16]. Among our study subjects, 85% of AD patients reported at least one ACE category [ACE(13) questionnaire] vs. 33% of controls [11]. AD patients with a positive history of lifetime suicide attempts endorsed a mean number of 4.6 ACEs categories [ACE(13) questionnaire]. This is in contrast to AD patients with a negative history of lifetime suicide attempts, who reported a mean number of 2.5 ACEs categories [ACE(13) questionnaire].

The diathesis-stress model of suicidal behavior claims that ACEs may be distal predictors of suicide attempts, creating vulnerability to adverse factors appearing later in life that are more proximal predictors of suicide attempts, i.e., loss of employment, health, or love. Our study confirmed that ACEs are in association relationship with lifetime suicide attempts in AD patients. Lifetime history of at least one suicide attempt was self-reported without information about reason, planning, the seriousness of declared attempts. Thus, the present study did not assess proximal predictors of suicide attempt. Paykel et al. [41] confirmed that suicide attempters reported four times as many life events in the 6 months prior to the attempt than controls. However, in our clinical sample, there was no significant difference between lifetime suicide attempters vs. non-attempters when basic socio-demographic variables were compared, including place of living, education, current employment, marital status, and living alone or with others. The cross-sectional design of our study did not provide data on mood or severity of alcohol drinking during the weeks or months prior to the suicide attempt, which all could be considered as risk factors of suicide attempts.

AD, depression, and anxiety disorders are frequently concomitant conditions [34]. Interestingly, alcohol intoxication, aggression, and impulsivity are more strongly associated with suicidal behavior than are depressive symptoms among AD patients [42, 43]. As reported by Kaplan et al. [44], postmortem blood alcohol content positivity was detected in about 36% of males and 28% of females that committed suicide between the years 2003 and 2011 in the US. In a Polish study of 162 suicide victims autopsied in the Department of Forensic Medicine between 2005 and 2006, 43% males and 31.3% females committed suicide under the influence of alcohol [45]. According to data from the Central Statistical Office of Poland [35], 620 of the 5405 individuals who completed suicide in Poland in 2016 were intoxicated with alcohol and 36 were intoxicated with psychoactive substances (of note, not all data on alcohol and psychoactive substances intoxication in completed suicides were available). However, there were no available data on if individuals who were intoxicated with alcohol prior to committing suicide were previously diagnosed with AD [35]. It is estimated that the risk of a suicide attempt is up to 60–120 times higher in AD patients than in individuals without a mental disorder [46]. Moreover, AD patients who attempt suicide under the influence of alcohol have been shown to choose more radical forms of suicide [47]. At the time of the study, our AD patients scored 27.2 ± 7.5 points (mean ± SD) on the AUDIT interview. Patients were not asked in a retrospective self-assessment if they were intoxicated with alcohol during the suicide attempt because we considered self-reported data on this issue to be potentially unreliable and would deliver only qualitative (rather than quantitative) data on alcohol intoxication. Wojnar et al. [10] found that 41 of 66 (66%) AD patients who confirmed a lifetime suicide attempt reported an impulsive character of the attempt, as they spent less than 30 min planning it. Kopera et al. [48] found that, although neuroticism and current depression symptoms severity differed significantly between the subgroup of AD lifetime suicide attempters vs. AD non-attempters among, mood regulation fully mediated the effects of neuroticism and current depression severity on lifetime suicide attempts. In the study by Gerhant et al. [38] AD patients who confirmed at least one-lifetime suicide attempt obtained higher scores in terms of aggression levels, harm avoidance, and self-directedness, and more frequently used the style of coping with stress that is based on avoidance and accepting the situation in comparison to AD patients with a negative lifetime history of suicide attempts. Few studies have examined generalized self-efficacy in AD patients [3, 11]. Task-oriented self-efficacy (drinking-refusal self-efficacy, self-efficacy to avoid suicidal action) but not generalized self-efficacy, was assessed in this clinical population [49, 50]. Generalized self-efficacy is considered to be a stable personality trait that is shaped in childhood and adolescence. In the study by Berent et al. [3], ACEs were found to be significantly related to generalized self-efficacy in AD patients, but explained only 3.2% of the variability in generalized self-efficacy, suggesting its multifactorial origin of final shape. It was found that stress-related adversities do not influence generalized self-efficacy in adulthood. For example, Jerusalem and Mittag [13] found, in a longitudinal study of migrants, that adverse events in adulthood did not significantly interfere with generalized self-efficacy. In their study, generalized self-efficacy was not significantly affected by the stress of migration, employment, and partnership status, and self-efficacy buffered the negative effects of stressful events [13]. Another study reported that generalized self-efficacy remained stable after severe somatic disorder in patients during a 6-month rehabilitation after myocardial infarction [51]. To our knowledge, this is the first study to confirm a role of generalized self-efficacy as a mediator in the effect of ACEs on lifetime suicide attempts in AD patients. Generalized self-efficacy was found to be a partial mediator of this relationship such that higher generalized self-efficacy was associated with a diminished effect of ACEs on the risk of suicide attempts. This finding provides an important clinical implication such that generalized self-efficacy should be considered as a target for psychotherapeutic interventions that aim to reduce the risk of suicide attempts in AD patients. Studies on therapy aimed at improving task-related self-efficacy showed that generalized self-efficacy may also be improved during the course of therapy. This pattern has been reported, for e.g., in studies of patients with mental disorders (*n* = 57) [52], in patients after spinal cord injury [53], parents of children with ataxia [54], and in college students [55]. However, there are no available data to indicate whether this improvement is long-lasting, despite the study in college students wherein the author observed that the improvement in generalized self-efficacy decreased after 3 months [55].

In the light of the current knowledge coming from GxE studies and epigenetics, there is still some contention regarding whether the position of genes as distal risks factor in the diathesis–stress model of suicidal behavior is appropriate. We proposed enlisting *FKBP5* rs1360780, *BDNF* rs6265, and *NRN1* rs1475157 as possible mediators of the relationship between ACEs and suicide attempts, and between ACEs and general self-efficacy; however, we found no association between the proposed SNPs and lifetime suicide attempts or general self-efficacy. Inconsistent data observed from studies on any SNPs as a risk factors for suicide may be due to several reasons, including (1) epigenetic differences between compared groups (e.g., promotor region methylation in the assessed gene); (2) obtaining statistical significance for a SNP only when assessed in haplotype, not in a single analysis; or (3) differences between the etiopathology of completed suicides (i.e., post-mortem studies) vs. suicide attempts (i.e., studies on suicide attempters). ACEs have a well-documented impact on epigenetic modifications, especially on methylation of the promoter region in genes related to the stress response and neuroplasticity. Gene expression regulation, independent of functional polymorphisms, may create a variety of clinical phenotypes and may be a cause of different results between studies that analyze early stress-related phenotypes based on SNPs [56]. Another possibility is that nucleotide polymorphisms documented as potentially protective when clinical phenotypes associated with ACEs is assessed may lose their protective role during ontogenesis due to promoter gene methylation. For instance, higher levels of methylation of the CpG island associated with the 5HTT promoter have been associated with increased risk of unresolved responses to loss or other trauma in carriers of the usually protective 5HTTLPR variant in the study by van IJzendoorn et al. [18]. However, it is important to underline that epigenetic modification may also be a primary, inherited modification of gene expression [57]. To summarize, genotypes that create a molecular background of susceptibility to an adverse environment (e.g., higher risk of suicide when one is raised in a dysfunctional household) may also create susceptibility to a beneficial environment if one is born in supportive milieu or translocated from an unsupportive environment in early childhood. Moreover, methylation of the promoter region may influence this molecular background for susceptibility additively or lift this effect. ACEs and homozygosity for the major T allele of *SSTR4* rs2567608 significantly raise the risk for lifetime suicide attempts in AD patients (i.e., homozygosity remained a significant predictor only in male AD patients) [11]. One study reported that the promoter region of *SSTR4*, which encodes the somatostatin receptor subtype 4, was more frequently methylated in AD patients than in controls; however, *SSTR4* promoter region methylation was not associated with the sum of ACEs categories or with any other factor analyzed in that study (term and course of labor, age and sex, nutritional habits, alcohol drinking severity, cigarette smoking) [20].

A haplotype TC composed of *FKBP5* rs1360780 and rs3800373 was found to be significantly associated with completed suicide in a general Japanese population [58]. A significant association of both TC haplotype and single T and C allele of *FKBP5* rs1360780 and rs3800373 and completed suicide was confirmed by Fudalej et al. [45]. Of 520 suicide victims analyzed by Fudalej et al. 54 had an available history of psychiatric disorders and/or AD. To our knowledge, the *FKBP5* rs1360780 T allele was found to be associated with suicide attempts only when analyzed in *FKBP5* haplotypes [17]. Our study also found no significant association between *FKBP5* rs1360780 and suicide attempt. It is noteworthy that *FKBP5* rs1360780 T allele carriers with a history of childhood abuse (i.e., emotional, physical, or sexual abuse and emotional and physical neglect were included) developed maladaptive emotional regulation patterns (i.e., rumination and catastrophizing) during adolescence, but only when assessed as a part of the haplotype *FKBP5* CATT composed of rs9296158, rs3800373, rs1360780, and rs9470080CATT [59]. We found no significant association between *FKBP5* rs1360780 and generalized self-efficacy in AD patients.

Carriers of the *BDNF* rs6265 66Met (A) allele and male sex were predictors of high lethality in suicide attempts in 120 patients with major depressive disorder (MDD) [60]. A post-mortem study conducted by Chojnicka et al. [29] found no association between *BDNF* rs6265 and suicide in 557 autopsied suicide victims in a heterogenous population, including 181 individuals with a confirmed history of psychiatric disorder (i.e., depression, schizophrenia, other unknown) and alcohol and/or drug addiction. Kohli et al. [61] found an association between *BDNF* rs6265 and suicide attempt in 394 MDD patients from the German population. Sarchiapone et al. [62] studied a sample of 170 patients with MDD and found a significantly higher risk of suicide attempts in patients reporting childhood emotional, physical, and sexual abuse, and in carriers of the *BDNF* rs6265 polymorphism variant (GA + AA). We found no association with *BDNF* rs6265, but to our knowledge, this is the first study that tested for an association between *BDNF* rs6265 and suicide attempts in a sample of AD patients.

Relatively few studies have examined the *NRN1* rs1475157 variant. A study on 410 carriers in non-clinical sample showed that *NRN1* rs1475157 GG homozygotes are significantly more prone to present sub-depressive symptoms than allele A carriers, and GG homozygotes have poorer cognitive performance in the Wisconsin Card Sorting Test and Phonemic Fluency as compared to allele A carriers. However, findings concerning cognition did not remain significant when correction for multiple testing was applied. Interestingly, carriers of both the GG genotype of *NRN1* rs1475157 and the *BDNF* rs6265 Met (A) allele presented with significantly more depressive symptoms [30]. To our knowledge, there are no studies that assess *NRN1* rs1475157 in suicide attempters or in those who have completed suicide. We found no association between *NRN1* rs1475157 and suicide attempts and generalized self-efficacy in AD patients.

## Conclusions

Generalized self-efficacy should be addressed as an intervention target for psychotherapy in AD patients as a possible strategy to reduce risk of suicide attempts in AD patients with a history of childhood maltreatment (over 85% of analyzed patients in this study). The list of questions about the 13 ACEs categories [ACE(13) questionnaire] may be considered as an interview tool to assess the presence of childhood maltreatment in AD patients.

Studies on larger samples are required to study our hypothesis about the possible mediating role of *FKBP5* rs1360780, *BDNF* rs6265, and *NRN1* rs1475157 in the relationship between ACEs and lifetime suicide attempts, and between ACEs and generalized self-efficacy in AD patients.

## Limitations

Results of the study should be interpreted with caution due to the following limitations:

First, our AD patients were recruited using the study inclusion and exclusion criteria from treatment-seeking AD patients, who are not representative of all AD patients (i.e., both treatment-seeking and untreated AD patients).

Next, we are limited by a possible recall bias due to the use of self-reported childhood victimization in a study with a cross-sectional design. This limitation is discussed in the “Discussion”. Nonetheless, the following efforts have been made to decrease the influence of recall bias on reporting ACEs and lifetime suicide attempts:

1. A 1:1 PSM was used to match controls and AD patients based on their age range, sex, and education. The PSM was set to reduce potential differences connected with these sociodemographic variables when the distribution of *FKBP5* rs1360780, *BDNF* rs6265, and *NRN1* rs1475157 allele and genotypes between AD patients and controls with low, medium, and high GSES levels as an outcome variable was analyzed (Supplementary Material Table 2A). The PSM was also implemented to match AD patient subgroups with a positive and negative lifetime history of a suicide attempts based on age range, sex, and education, and also to reduce recall bias in self-reports of a suicide attempt (Supplementary material Table 3A). All calculations that were significant in the primary analysis (Tables 2, 3) remained significant with the PSM, and all calculations that were not significant remained not significant.
2. Patients with psychiatric comorbidity requiring current treatment were excluded from the study. As reviewed by Norman et al. [63], individuals with adjustment disorder are more prone to recall or disclose exposure to abuse and neglect. However, at least with respect to child sexual abuse, evidence suggests moderate to good consistency of reports over time, and biases are likely towards under-reporting rather than over-reporting of abuse [63].

A history of lifetime suicide attempts was identified with a single question. Data about various characteristics of the suicide attempt (i.e., recency and seriousness) are not available.

The study sample of AD patients and controls is relatively small. Homozygotes for minor *BDNF* rs6265 and *NRN1* rs1475157 alleles were seen only in 1–5 controls or AD patients, and ten controls or AD patients for *FKBP5* rs1360780. Thus, negative results on the association between the assessed genotypes (*FKBP5* rs1360780, *BDNF* rs6265, and *NRN1* rs1475157) and lifetime suicide attempts and generalized self-efficacy should be interpreted cautiously. Further studies on larger samples are required. However, our study is the first to address these aims in AD patients and creates a theoretical foundation for future studies on this issue.

## Electronic supplementary material

Below is the link to the electronic supplementary material.

Supplementary material 1 (DOCX 28 kb)
