# Correction to: The role of childhood adversities, *FKBP5*, *BDNF*, *NRN1*, and generalized self-efficacy in suicide attempts in alcohol-dependent patients

**DOI:** 10.1007/s43440-021-00277-5

**Published:** 2021-06-22

**Authors:** Dominika Berent, Bożena Szymańska, Dominika Kulczycka-Wojdala, Marian Macander, Zofia Pawłowska, Marcin Wojnar

**Affiliations:** 1 Masovian Regional Psychiatric Hospital Drewnica, Ząbki, Poland; 2 Central Scientific Laboratory, Medical University of Lodz, Lodz, Poland; 3 Aviation Patophysiology and Safety Flight Department, Military Institute of Aviation Medicine, Warsaw, Poland; 4 Department of Psychiatry, Medical University of Warsaw, Warsaw, Poland; 5 Department of Psychiatry, University of Michigan, Ann Arbor, MI USA

## Correction to: Pharmacological Reports (2020) 72:730–743 10.1007/s43440-020-00080-8

The article “The role of childhood adversities, *FKBP5, BDNF, NRN1*, and generalized self-efficacy in suicide attempts in alcohol-dependent patients”, written by Dominika Berent, et al., was originally published electronically on the publisher’s internet portal on 10 March 2020 without open access. With the author’ decision to opt for Open Choice the copyright of the article changed on 30 April 2021 to © Author(s) 2020 and the article is forthwith distributed under a Creative Commons Attribution 4.0 International License, which permits use, sharing, adaptation, distribution and reproduction in any medium or format, as long as you give appropriate credit to the original author(s) and the source, provide a link to the Creative Commons licence, and indicate if changes were made. The images or other third party material in this article are included in the article’s Creative Commons licence, unless indicated otherwise in a credit line to the material. If material is not included in the article’s Creative Commons licence and your intended use is not permitted by statutory regulation or exceeds the permitted use, you will need to obtain permission directly from the copyright holder. To view a copy of this licence, visit http://creativecommons.org/licenses/by/4.0.

The original article has been corrected.

