# Supplementary material for: The role of childhood adversities, FKBP5, BDNF, NRN1, and generalized self-efficacy in suicide attempts in alcohol-dependent patients
Source: Pharmacol Rep. 2020 Mar 10;72(3):730–43. doi: 10.1007/s43440-020-00080-8 (PMC8217039; doi:10.1007/s43440-020-00080-8)
Supplement: Supplementary file 1 — Supplementary material 1 (DOCX 28 kb) [file 43440_2020_80_MOESM1_ESM.docx]

Supplementary Material

The PSM (propensity score matching) with 1:1 matching controls and AD patients based on their age range, sex, and education was set to reduce possible differences connected with these sociodemographics when the distribution of *FKBP5* rs1360780, *BDNF* rs6265, and *NRN1* rs1475157 allele and genotypes in AD patients and the controls divided by low, medium, and high GSES outcome was analyzed (Table 2A). The distribution of *FKBP5* rs1360780, *BDNF* rs6265, and *NRN1* rs1475157 allele and genotypes in same age, sex and education matched AD patients and control subjects was additionally checked (Table 1A). The PSM was also implemented to match AD patient subgroups with positive and negative lifetime history of a suicide attempt based on age range, sex, and education, and also to reduce recall bias in self-reports of a suicide attempt (Table 3A). All calculations that were significant in primary analysis (Tables 1-3) remained significant and all calculations that were not significant, remained not significant.

Table 1 A. The distribution of *FKBP5* rs1360780, *BDNF* rs6265, and *NRN1* rs1475157 allele and genotypes in AD patients (n=75) and control subjects (n=75) [the propensity score matching with 1:1 matching controls and AD patients based on their age range, sex, and education]

|  |  | Controls (n = 75) | | AD Patients (n = 75) | | Chi^2^ | p |
| --- | --- | --- | --- | --- | --- | --- | --- |
|  |  | n | % | n | % |  |  |
|  | T/T | 7 | 9.3 | 3 | 4.0 |  |  |
| *FKBP5* | C/T | 34 | 45.3 | 29 | 38.7 | 3.049 | 0.218^2^ |
|  | C/C | 34 | 45.3 | 43 | 57.3 |  |  |
| rs1360780 |  |  |  |  |  |  |  |
|  | C | 68 | 90.7 | 72 | 96.0 | 1.714 | 0.190^2^ |
|  |  |  |  |  |  |  |  |
|  | T | 41 | 54.7 | 32 | 42.7 | 2.162 | 0.142^2^ |
|  | A/A | 2 | 2.7 | 1 | 1.3 |  |  |
| *BDNF* | G/A | 17 | 22.7 | 22 | 29.3 |  | 0.589^1^ |
|  | G/G | 56 | 74.7 | 52 | 69.3 |  |  |
| rs6265 |  |  |  |  |  |  |  |
|  | G | 73 | 97.3 | 74 | 98.7 |  | >0.999^1^ |
|  |  |  |  |  |  |  |  |
|  | A | 19 | 25.3 | 23 | 30.7 | 0,529 | 0.586^2^ |
|  | G/G | 2 | 2.7 | 1 | 1.3 |  |  |
| *NRN1* | A/G | 13 | 17.3 | 19 | 25.3 |  | 0.485^1^ |
|  | A/A | 60 | 80.0 | 55 | 73.3 |  |  |
| rs1475157 |  |  |  |  |  |  |  |
|  | A | 73 | 97.3 | 74 | 98.7 |  | >0.999^1^ |
|  |  |  |  |  |  |  |  |
|  | G | 15 | 20.0 | 20 | 26.7 | 0.932 | 0.334^2^ |

p – level of statistical significance with Bonferroni correction;

^1^–Fisher’s exact test; ^2^ – Chi square test AD – alcohol-dependent

Table 2A. The distribution of *FKBP5* rs1360780, *BDNF* rs6265, and *NRN1* rs1475157 alleles in AD patients (n=75) and the controls (n=75) divided by low, medium, and high GSES outcome [the propensity score matching with 1:1 matching controls and AD patients based on their age range, sex, and education].

|  |  | GSES |  | low | medium | | high |  |  |
| --- | --- | --- | --- | --- | --- | --- | --- | --- | --- |
|  |  |  | n | % | n | % | n | % | p |
|  |  |  |  |  |  |  |  |  |  |
|  | AD patients | C | 11 | 100.0 | 51 | 100.0 | 12 | 92.3 | 0.159^2^ |
|  |  | T | 1 | 9.1 | 15 | 29.4 | 7 | 53.8 | 0.067^1^ |
| *BDNF* |  |  |  |  |  |  |  |  |  |
|  | Controls | C |  |  | 29 | 96.7 | 44 | 97.8 | >0.999^1^ |
| rs6265 |  | T |  |  | 5 | 16.7 | 14 | 31.1 | >0.999^1^ |
|  |  |  |  |  |  |  |  |  |  |
|  | AD patients vs | C |  |  | 0.370^1^ | | 0.401^1^ | |  |
|  | Controls | T |  |  | 0.199^2^ | | 0.191^1^ | |  |
|  | AD patients | C | 10 | 90.9 | 50 | 98.0 | 12 | 92.3 | 0.238^1^ |
|  |  | T | 4 | 36.4 | 22 | 43.1 | 6 | 46.2 | 0.882^1^ |
| *FKBP5* |  |  |  |  |  |  |  |  |  |
|  | Controls | C |  |  | 27 | 90.0 | 41 | 91.1 | >0.999^1^ |
| rs1360780 |  | T |  |  | 15 | 50.0 | 26 | 57.8 | 0.507^2^ |
|  |  |  |  |  |  |  |  |  |  |
|  | AD patients vs | C |  |  | 0.141^1^ | | >0.999^2^ | |  |
|  | Controls | T |  |  | 0.549^2^ | | 0.535^2^ | |  |
|  | AD patients | A | 11 | 100.0 | 50 | 98.0 | 13 | 100.0 | >0.999^1^ |
|  |  | G | 2 | 18.2 | 16 | 31.4 | 2 | 15.4 | 0.449^1^ |
| *NRN1* |  |  |  |  |  |  |  |  |  |
|  | Controls | A |  |  | 29 | 96.7 | 44 | 97.8 | >0.999^1^ |
| rs1475157 |  | G |  |  | 7 | 23.3 | 8 | 17.8 | 0.556^2^ |
|  |  |  |  |  |  |  |  |  |  |
|  | AD patients vs | A |  |  | >0.999^1^ | | >0.999^1^ | |  |
|  | Controls | G |  |  | 0.438^2^ | | >0.999^1^ | |  |

p – level of statistical significance with Bonferroni correction; ^1^ – Fisher’s exact test; ^2^ – Chi square test; GSES – generalized self-efficacy scale; AD – alcohol-dependent

Table 3A. Comparison of AD patients with a negative vs. positive lifetime history of at least one suicide attempt [the propensity score matching with 1:1 matching controls and AD patients based on their age range, sex, and education].

|  |  | ^Lifetime history of at least 1 suicide attempt in AD patients^ | |  |
| --- | --- | --- | --- | --- |
|  |  | negative  n = 37 | positive  n = 37 | *p* |
| Place of living n(%) | Village | 0 (0) | 5 (13.5) | 0.054^1^ |
|  | Urban area | 37 (100.0) | 32 (86.5) |  |
| Occupational status n(%) | Employed | 10 (27.8) | 10 (27.0) | 0.878^1^ |
|  | Unemployed | 21 (58.3) | 24 (64.9) |  |
|  | Retired | 1 (2.8) | 1 (2.7) |  |
|  | On pension | 4 (11.1) | 2 (5.4) |  |
| Marital status n(%) | Single | 21 (56.8) | 15 (40.5) | 0.036^1^ |
|  | Married | 9 (24.3) | 5 (13.5) |  |
|  | Divorce | 3 (8.1) | 13 (35.1) |  |
|  | widowed | 4 (10.8) | 4 (10.8) |  |
| Living status n(%) | Alone | 18 (48.6) | 19 (51.4) | 0.465^1^ |
|  | With family | 16 (43.2) | 12 (32.4) |  |
|  | With partner | 3 (8.1) | 6 (16.2) |  |
| ACE Study Score  Mean ± SD |  | 2.22 (2.03) | 3.86 (2.42) | 0.003^3^ |
| ACE(13)  Mean ± SD |  | 2.54 (2.23) | 4.73 (2.67) | 0.001^3^ |
| GSES  Mean ± SD |  | 29.65 (5.29) | 25.27 (6.63) | 0.004^3^ |
| *FKBP5*  rs1360780  allele  genotypes  n (%) | T/T | 3 (8.1) | 2 (5.4) | 0.393^1^ |
|  | C/T | 13 (35.1) | 19 (51.4) |  |
|  | C/C | 21 (56.8) | 16 (43.2) |  |
|  | A | 34 (91.9) | 35 (94.6) | >0.999^1^ |
|  | G | 16 (43.2) | 21 (56.8) | 0.245^2^ |
| *NRN1* | G/G | 2 (5.4) | 0 (0) |  |
| rs1475157 | A/G | 6 (16.2) | 10 (27.0) | 0.276^1^ |
| allele | A/A | 29 (78.4) | 27 (73.0) |  |
| genotypes | A | 35 (94.6) | 37 (100.0) | 0.493^1^ |
| n (%) | G | 8 (21.6) | 10 (27.0) | 0.588^2^ |
| *BDNF* rs6265 | A/A | - | - |  |
| allele | G/A | 12 (32.4) | 11 (29.7) | 0.802^2^ |
| genotypes | G/G | 25 (67.6) | 26 (70.3) |  |
| n (%) | C | 37 (100.0) | 37 (100.0) |  |
|  | T | 12 (32.4) | 11 (29.7) | 0.802^2^ |

p – level of statistical significance (bold values mean a statistical significance according to Bonferroni correction (p<0.005))

^1^ - U Mann-Whitney test; ^2^ – Chi square test; ^3^ - Fisher’s exact test - bold values mean a statistical significance; ACEs – adverse childhood experiences; *ACE Study questionnaire scoring; **ACE (13) questionnaire scoring; AD – alcohol-dependent; GSES – generalized self-efficacy scale; SD- standard deviation
